# Observations on the Advantages of Exposing Wounds to the Air after Capital Operations; with Some Remarks upon the Removal of Scirrhous Tumours from the Breast. Communicated in a Letter to Samuel Bard, M.D. President of the College of Physicians and Surgeons

**Published:** 1814-06

**Authors:** David Hosack

**Affiliations:** Professor of the Theory and Practice of Physic and Clinical Medicine in the College of Physicians and Surgeons, New York


*For the Medical and Physical Journal.*

*Observations on the Advantages of exposing Wounds to the Air after capital Operations; with some Remarks upon the Removal of Scirrhous Tumours from the Breast. Communicated in a Letter to Samuel Bard, M.D. President of the College of Physicians and Surgeons.* By David Hosack, M.D. Professor of the Theory and Practice of Physic and Clinical Medicine in the College of Physicians and Surgeons, New York.

Dear Sir, *New York, June* 29, 1813.

MANY years since, you related to me the case of a patient whose limb you amputated, in which you found great difficulty in restraining the hemorrhage, but in which you at last succeeded, by keeping the stump exposed to the air a considerable length of time after the removal of the limb.

Although hemorrhage has ever constituted one of the most formidable embarrassments attendant upon surgical operations, and much ingenuity has been exercised in devising a great variety of astringent and other applications, for the suppression of it, yet this practice has not, to my knowledge, sufficiently attracted the attention of surgeons. Mr. Cooper, one of the latest and most respectable writers on surgery, incidentally notices the styptic effects of cold air, in promoting the contraction of the vessels; but he appears to introduce the remark for the purpose of showing, that no dependance is to be placed upon it; for he adds, that, "upon the wound being dressed, and the patient placed in bed, not an hour elapses before the renewal of the hemorrhage necessitates us to remove the dressings."*

---

* See Cooper's Dictionary of Practical Surgery, Dorsey's edition, vol. i. p. 438.

In

In the following case, which, from the advanced age of the patient, the duration of the disease, his debilitated habit of body, and other circumstances, was of all others the most unpromising, the exposure of the limb was attended with the most beneficial effects; as such, I communicate it to you, believing that this plan of restraining and of preventing the return of hemorrhage, may prove a valuable auxiliary in the hands of others under similar circumstances.

General William Crane, now resident in the vicinity of Elizabeth Town, New Jersey, was a lieutenant in one of the New York regiments, at the commencement of the revolutionary war, and was at the taking of St. John's, (Canada,) in the year 1775. While engaged in laying a platform on the batteries, he was struck by a shell on the inside of the leg, a few inches above the ankle joint. Although the blow was violent, and injured the tibia as well as the soft parts, such was the fervor of his mind, that he totally disregarded it for twenty-four hours, when inflammation ensued, which ended in ulceration and a caries of the bone, that continued until the removal of his limb. His system has been greatly debilitated by the discharge from this long-continued and extensive ulceration; but possessing a strong constitution, an equal and amiable temper of mind, and observing the greatest regularity and temperance in his mode of living, he has, in all other respects, enjoyed a considerable share of health, notwithstanding the drain he has sustained for nearly forty years.

The following extract of a letter from his attending physician, Dr. Isaac Morse, of Elizabeth Town, contains the most important circumstances which took place prior to the operation.

"DEAR SIR, *Elizabeth Town, April* 29, 1813.

"I have been conversant with General William Crane, and have had knowledge of the ulcer on his leg upwards of twenty-five years; he has been, and now is, one of the most temperate men I ever knew; he spared no time, pains, or expense, to obtain relief, but has found none. On the 12th day of December last I visited him. At that time he was under the care of Dr. Cook of Bound Brook. We found the ulcer very large and deep, a great part of the tibia bare and carious, with a considerable hemorrhage from the capillary vessels, together with the usual discharge from the ulcer, by which the General had become very much reduced. The course or channel of the tibial artery lay exposed to view. We were of opinion, that the artery would soon be destroyed, and advised every precaution to be taken, should it happen in our absence; but very fortunately for him, when

Dr. Cook was dressing the ulcer, on the 19th day of December last, in my presence, the artery burst with such a violent hemorrhage, that with all our attention he lost at least twenty ounces of blood, which added much to his debility; but the artery was taken up and secured by a ligature. A considerable swelling of the leg and foot took place below the ligature, and continued for some time after, when the ulcer put on a more healthy appearance; those parts which were not irritated by the carious bones soon began to heal, and the General to recover his strength. I was unable to attend him on account of a fracture of my arm. Dr. Cook attended as usual; in about seven weeks I again visited him, found him in better health, and his strength greatly improved, the ulcer as when I left him, with the tibia almost destroyed. I then, for the first time, advised him to have his leg amputated, before which time the great debility of his body, by reason of the loss of so much blood, rendered the operation, in my opinion, very doubtful.

I am, dear Sir, with great regard, your's, &c.

ISAAC MORSE."

A consultation of the physicians of Elizabeth Town and its vicinity being held, it was decided, that unless the leg was immediately removed, he must inevitably sink under the discharge he then sustained.

On the 4th day of April last, I was requested to visit General Crane, for the purpose of performing the operation, if, upon another consultation, an amputation of the limb should still be thought expedient. I found the patient very much emaciated, his leg swelled and œdematous, the ulcer very extensive, but of a healthy appearance, and discharging well-formed matter.

Under existing circumstances, we were unanimously of opinion, that unless the operation should be immediately performed, the General must sink in a very few weeks, and that his only resource was in the loss of his limb. The result being made known to him, with his characteristic firmness he at once yielded to our advice.

I immediately proceeded to the operation, which the majority of those present thought it advisable should be performed below the knee.

Upon the first incision being made through the integuments, a very large quantity of serum was discharged from the cellular membrane; upon dividing the muscles, these also were found in a very pale and flaccid state, exhibiting very little of that retractive power which usually takes place upon amputation. The bones were then divided by slow and steady movements of the saw, so as to prevent their edges from splintering, an accident which frequently occurs in consequence of the rapidity and violence with which surgeons usually conduct this part of the operation, and which seldom fails to end in a tedious caries of the bones so splintered.

To my great surprise, it became necessary to secure six large arteries, which, probably, had become thus enlarged in consequence of the anterior tibial artery having been tied some months before, as stated by Dr. Morse. The tourniquet being loosened, the blood still continued to flow profusely from the whole surface of the stump, even from the vessels of the bones.

As it was not possible to detect any particular arteries from whence the hemorrhage proceeded, I resolved to restrain the discharge, at first, by moderately tightening the tourniquet for a few minutes, and afterwards to leave the stump exposed to the air until the bleeding should totally cease. I accordingly directed the persons present, excepting my assistants, to withdraw into another room; the windows to be raised, and some wine to be given to the patient, who was somewhat faint and exhausted. The stump was then exposed to a stream of fresh cool air; the hemorrhage immediately abated, and in a short time totally ceased, when the tourniquet was entirely taken from the limb.

After thus exposing the stump to the air about half an hour, (and, as I find, exposing myself to the censure of some of the by-standers, who, with watch in hand, counted every minute that passed,*) I proceeded by means of a sponge and warm water, to remove all the coagulated blood from its surface; no further hemorrhage ensued: the wound was then dressed in the ordinary manner; the patient conveyed to his bed; the limb lightly covered with a sheet, and a tourniquet loosely placed upon the thigh. An anodyne of fifty drops of liquid laudanum was then administered, and a direction given to repeat the same quantity at night, if pain, spasm, or great restlessness should render it necessary.

* Having been severely censured by some of the *surgeons* of the village, for keeping my patient upwards of forty minutes on the operating table, I feel myself constrained to ask the same question Mr. Bromfield, of St. George's Hospital, addressed to a pupil who pulled out his watch at the moment the doctor was about to commence his operation: "What, Sir, do you intend to measure the movements of my hand, as you would a horse's feet?" adding, "Sir, let me tell you, that that operation is always *soon* enough done that is *well enough* done." Macbeth, when about to commit murder, says,

"——————— 'twere *well*

It were done *quickly*."

But this should never be the surgeon's motto.

Upon

Upon the following day, I had the pleasure of receiving a line from Dr. Morse, informing me that our patient had passed a very comfortable night, without hemorrhage, pain, spasm, or any untoward symptoms. On Thursday, the 8th, I again visited General Crane, and dressed his limb. Upon removing the dressings, which had been previously moistened by spirits, the wound, to our surprise, was united throughout its whole extent, excepting at the lower angle and another aperture through which the ligatures were passed, and discharging a well-digested matter, without the least tinge of blood. Upon the seventh day after the operation, the ligatures came away; the patient was then put upon the use of bark and wine; the limb daily bathed with spirits at each dressing; the wound at the same time excited by an injection, consisting of a solution of the sulphate of zinc in rain water, in the proportion of four grains to the ounce; the surface of the wound covered with dry lint and a light compress of linen; and the whole retained by a flannel roller.

By this general and local use of stimulants and tonics, and a corresponding attention to the diet and regimen of our patient, in about four weeks his wound was closed, and his health so far restored, that he has been enabled to resume the direction of his farm, with the prospect of adding many happy years to his life, to the great gratification of his family and his numerous friends.

Whatever may be the opinion we form of the process nature employs in checking hemorrhage, this case, under all the circumstances attendant upon it, is an evidence of the good effects of exposing a bleeding wound to the air.

Whether we believe the hemorrhage to be restrained by the formation of an internal* or an external coagulum, by the effusion and coagulation of blood in the surrounding cellular membrane,† by the retraction‡ of the artery, a constriction§ of the circular fibres of its extremity, or an effusion of lymph‖ from the divided vessels and the inflamed vasa vasorum,—in either case, the long-continued application of a stream of fresh air upon the part, is well calculated to prevent the evil to be apprehended; at the same time that the stimulant effects of the air, in cases like the present, will probably not a little continue to excite the degree of inflammation in the wound which is necessary to accomplish the union by first intention. Its benefit, in this respect, was very apparent; for such was the enfeebled state of the General's constitution,

* Petit. † J. Bell. ‡ Pouteau.

§ Morand, Gooch, White, Kirkland, &c. ‖ Jones.

and, and, as has already been remarked, so relaxed were the parts themselves, that an immediate union of the sides of the wound was scarcely to have been expected without the aid of some local additional stimulus.

During your last visit to this city, in the early part of this month, you also had an opportunity of witnessing the success of this practice in restraining a very tedious and troublesome hemorrhage, which took place after an amputation of the breast. In that case, as is usual after that operation, although I had secured ten arteries by ligature, the discharge of blood was very considerable from the whole surface; but after remaining exposed for half an hour, the hemorrhage having totally ceased during the greater part of that time, the wound was dressed, and as in the former case, upon dressing it four days after, its sides were entirely closed, excepting that part through which the ligatures were passed. I have great pleasure in adding, that the patient has perfectly recovered.

In this too, as in the former case, the healing process was greatly promoted by the free use of bark and wine, a generous diet, daily bathing the parts with spirits, and by injecting into the wound a solution of the sulphate of zinc. I mean that these advantages were obtained after the first inflammatory stage had subsided, and the purulent secretion had taken place; for in the treatment of wounds, as well as in that of ulcers, surgeons cannot too carefully distinguish between these two stages, which may with great propriety be denominated the *active* and the *passive* stages of an ulcer or a wound.

As I have adverted to the extirpation of the breast, I cannot close this letter without mentioning the benefits which I have derived upon this subject from the valuable cases recorded by Professor Richter;* and which do not appear to be sufficiently known and appreciated in this country. I refer more particularly to his treatment of scirrhous and cancerous breasts, in which he recommends the removal of the entire breast, even though the disease may be limited to a single gland.

In the first case of this nature in which I was called upon to operate, as early as the year 1795, the disease was confined to a small part of the breast, the remainder being apparently in its natural and healthy state; it was the opinion of all present that it was only necessary to remove the gland

---

* Medical and Surgical Observations, by Aug. Gottlieb Richter, M.D. Professor of Medicine in the University of Goettingen, &c.

affected:

affected: this was accordingly done; but, within three months after, the other portion of the breast became diseased, when a second operation was rendered necessary for its total removal. Similar cases have also occurred in the practice of others, and in some instances the renewal of the disease, the constitution having been enfeebled by the first operation, has ended fatally. Since that period, I have uniformly performed this operation by removing the whole glandular part of the breast, but retaining the integuments, except when they also have partaken of the disease; and in every case so operated upon, the patient has afterwards remained perfectly free from any return of the complaint.

This fact leads to two important conclusions: 1st. That in most instances the scirrhous or cancerous affection of the breast is a local disease, and not a constitutional one, as many physicians have supposed. 2dly. That in those cases where only the part of the breast was removed, and the remainder had become subsequently affected, the latter disease had probably been induced by the inflammation occasioned by the operation in removing the first, and not from any vice of the whole system; for in those cases which I have witnessed, the secondary disease commenced during the existence of the inflammation which the operation had excited, and before the wound had entirely healed. I am, dear Sir,

With great respect and esteem, your's,

DAVID HOSACK.

*Samuel Bard, M.D. &c.*

